# The Regulations of Essential WalRK Two-Component System on *Enterococcus faecalis*

**DOI:** 10.3390/jcm12030767

**Published:** 2023-01-18

**Authors:** Junqi Zhang, Rong Fang, Qi Peng, Shizhou Wu, Lei Lei

**Affiliations:** 1 Orthopedic Research Institute, Department of Orthopedics, West China Hospital, Sichuan University, Chengdu 610017, China; 2 West China School of Public Health and West China Fourth Hospital, Sichuan University, Chengdu 610017, China; 3 West China Hospital of Stomatology, Sichuan University, Chengdu 610017, China

**Keywords:** *Enterococcus faecalis*, two-component system, WalRK, biofilm, antibacterial strategies

## Abstract

*Enterococcus faecalis* (*E. faecalis*) is a Gram-positive, facultative anaerobic bacterium that is highly adaptable to its environment. In humans, it can cause serious infections with biofilm formation. With increasing attention on its health threat, prevention and control of biofilm formation in *E. faecalis* have been observed. Many factors including polysaccharides as well as autolysis, proteases, and eDNA regulate biofilm formation. Those contributors are regulated by several important regulatory systems involving the two-component signal transduction system (TCS) for its adaptation to the environment. Highly conserved WalRK as one of 17 TCSs is the only essential TCS in *E. faecalis*. In addition to biofilm formation, various metabolisms, including cell wall construction, drug resistance, as well as interactions among regulatory systems and resistance to the host immune system, can be modulated by the WalRK system. Therefore, WalRK has been identified as a key target for *E. faecalis* infection control. In the present review, the regulation of WalRK on *E. faecalis* pathogenesis and associated therapeutic strategies are demonstrated.

## 1. Introduction

*Enterococcus faecalis* (*E. faecalis*) is a Gram-positive, facultative anaerobic bacterium, which almost serves as normal colonizing bacteria in the gastrointestinal tracts of humans and is generally related to commensal life in the gastrointestinal tract [1]. *E. faecalis* has strong environmental adaptability. The abilities to form robust and mature biofilms [2,3,4] makes *E. faecalis* able to survive in scarce nutrition and resist extreme alkaline pH values [5,6,7,8]. With biofilm formation, *E. faecalis* can also cause serious infection. As one of the most common pathogens in hospital infections, *E. faecalis* leads to various opportunistic infections [9,10,11,12,13] such as urinary tract infections, bacteremia, prosthetic joint infections, abdominal-pelvic infections, endocarditis [14,15,16], periodontal disease, and endodontic infections [17,18]. Despite its pathogenicity, *E. faecalis* is also a probiotic and is often found as part of the autochthonous flora in a variety of fermented foods, namely cheeses and sausages [19,20,21,22,23].

Among the regulatory systems in *E. faecalis* [24], the two-component signal transduction system (TCS) is one of the signal pathways necessary for bacteria to adapt to the environmental changes, which helps bacteria to perceive and respond to external signals rapidly. A TCS consists of two basic components: histidine kinase (HK), which is anchored to the cell membrane, and intracellular response regulators (RRs). The HK responds to the environmental stimulus by auto-phosphorylating on a conserved histidine residue in the cytoplasmic domain. Then, the phosphate group is transferred to an aspartate residue on the partner RR. Through affinity for target promoter DNA, RR effects a corresponding modulation of transcription and elicits a cellular response to the environmental stimuli [25,26].

*E. faecalis* includes 17 members of two-component systems, in which the CroRS system is required for intrinsic β-lactam resistance [27,28]. The β-lactams are structural analogs of the D-Ala4-D-Ala5, an extremity of peptidoglycan precursors, and act as suicide substrates of the D, D-transpeptidases that catalyze the last cross-linking step of peptidoglycan synthesis [29]. The CroS HK activates in response to the cell wall damage induced by antimicrobial effects, and CroR upregulates an alternative penicillin-binding protein (PBP), carrying out transpeptidase activity with a low-binding affinity for β-lactams [30]. By enhancing the release of nucleic acids into the biofilm matrix, the two-component Fsr-QS (quorum sensing) system controls the production of extracellular gelatinase and contributes to the biofilm formation [31]. The *fsr* regulatory locus is comprised of three genes, designated as FsrA, FsrB, and FsrC, which are necessary for positive regulation of the virulence-associated proteases Gelatinase E (GelE) and serine protease (SprE) [32,33]. FsrC is a histidine kinase that senses extracellular accumulation of a peptide lactone encoded at the C-terminus of the FsrB protein. The sensing of FsrC leads to activation of the response regulator and transcription factor FsrA [34]. In addition, the Fsr system positively regulates degradation of important immunopeptides, exerts an inhibitory effect against the complement system, and performs a critical role in overcoming the immune systems that are inherent to human serum [34,35,36]. Glycopeptide antibiotics vancomycin (VM) and teicoplanin (TE) inhibit the extracellular steps of bacterial peptidoglycan synthesis by binding to the C-terminal D-alanyl-D-alanine (D-Ala-D-Ala) residues of cell wall precursors. Synthesis of modified peptidoglycan precursors terminating in D-lactate, glycopeptide such as peptidoglycan precursor UDP-N-acetylmuramyl-L-Ala-γ-D-Glu-L-Lys-D-Ala-D-lactate (UDP-MurNAc-pentadepsipeptide) makes a more than 1000-fold decreased affinity for glycopeptides resistance. In *E. faecalis*, the TCS VanSRB regulates an immediately downstream promoter and contributes to VanB-type vancomycin resistance. Vancomycin is identified as an inducer for the VanSBRB activation [27,37]. Among all 17 TCSs, walRK is the only essential TCS for *E. faecalis,* which determines the viability of stains, indicating that the survival of bacteria can be significantly affected by the knockout of *walRK* [26].

The WalRK (also named YycFG or VicRK) TCS signaling pathway is one of the most widely distributed TCS systems, highly conserved in most low-G+C Gram-positive bacteria [38]. The system has been reported as necessary for several closely related pathogens including *Staphylococcus aureus* [39], *Streptococcus pneumoniae* [40], and *Bacillus subtilis* [41]. The core of the WalRK TCS consists of HK (WalK) on the cell surface and an intracellular RR (WalR) [25]. The WalK histidine kinases contain HAMP (a domain present in histidine kinases, adenylyl cyclases, methyl-accepting proteins, and phosphatases) and PAS (Per-Arnt-Sim) sensing domains, which promote two-way conformational communication between the input and output domains signaling proteins, along with histidine phosphotransfer (HisKA) and kinase catalytic (HATPase) domains [42,43,44,45]. The amino acid sequences of the receiver and effector domains of WalR are highly conserved and belong to the OmpR family of response regulators [44,45,46]. In addition to *walR* and *walK*, *yycH*, *yycI,* and *yycJ* are also included in the *E. faecalis* WalRK TCS [47,48]. The auxiliary proteins YycHI play roles in signaling through the WalK [45]. Although the cytoplasmic, conserved YycJ auxiliary protein contains a putative metal binding site in a β-lactamase fold [49], its functions are currently unknown [45]. In this review, we investigate the role of the WalRK system in the metabolism of *E. faecalis*, including biofilm formation, cell wall construction, drug resistance, and resistance to the host immune system. In addition, potentially therapeutic strategies associated with the WalRK TCS are noted for clinical infections administration.

## 2. Material and Methods

Based on searching the PubMed database with terms “WalR, WalK, two-component system, *Enterococcus faecalis*, regulation, and treatment”, we conduct a literature search. The inclusion criteria included: (1) original studies or reviews on related topics; (2) published in English; (3) full-text. The exclusion criteria included: (1) abstracts from conferences and (2) document cannot be downloaded. The search in PubMed yielded 426 articles and, after exclusion, 122 were included in our literature manager. To supply our possibly ignored information, associated references in the already included literature were also detected.

## 3. WalKR Two-Component Signal Transduction System and Resistance of *Enterococcus Faecalis*

### 3.1. Regulation of E. faecalis on Biofilm Formation

*E. faecalis* is characterized by its ability to survive in a high-sodium-chloride concertation and tolerate both acid and alkaline conditions. With the widespread use of antibiotics, *E. faecalis* as a pathogenic bacterium has gradually emerged with antibiotic resistance. Those adaptive capabilities are closely related to the *E. faecalis* biofilm formation.

Biofilm is mainly developed by packed microcolonies and extracellular polymeric substances (EPSs). Multiple genes have been identified to play an important role in biofilm biogenesis. Genes encode proteinaceous adhesins such as LPxTG surface proteins, autolysins, and glycolipids, contributing to intercellular adhesion in biofilm formation. EF3314 is a putative surface-exposed antigenic protein in *E. faecalis*. It is considered as an adhesin for biofilm formation.

Polysaccharides, as well as autolysins and proteases, are thought to contribute to EPS production, and therefore affect biofilm maturation. Gene cluster *epa* (enterococcal polysaccharide antigen) is involved in the biosynthesis of cell-wall-associated polysaccharides. In *E. faecalis*, the *epa* gene cluster encodes enzymes and transporters involving synthesis of nucleotide sugar precursors in the cytoplasm, formation and polymerization of repeating units, and export to the cell surface, which contribute to biofilm formation, resistance to enterococcal polysaccharide antigen (PMN) killing, and virulence [50]. 

eDNA is an important component of the extracellular matrix of bacterial biofilms, providing structural stability to the biofilm and protection against antimicrobials. Murein hydrolases, also referred to as autolysins, have been implicated in biofilm production [51]. In *E. faecalis*, autolysins such as AtlA, AtlB, and AtlC are involved in eDNA release. AtlA is identified as an N-acetylglucosaminidase [52]. The synergistical action with two additional peptidoglycan hydrolases AtlB and AtlC DNA regulates the release of eDNA [53]. The deletion of *atlA* not only delays the biofilm formation but also inhibits cells division and reduces the rate of cellular lysis [53].

Via proteolytic processing of the accumulation-associated protein, bacterial proteases contribute to biofilm formation [54,55]. Two proteases produced by *E. faecalis* such as GelE and SprE have been shown to regulate bacterial autolysis as well as eDNA release and, thus, contribute to *E. faecalis* biofilm formation, playing important roles in the pathogenesis of various diseases, including endophthalmitis, peritonitis, endocarditis, and orthopedic implant infections [56]. Regarding orthopedic implant infections, *E. faecalis* with high gelatinase production is a prevalent isolate [57]. The quorum-sensing Fsr two-component system is comprised of three genes involving *fsrA*, *fsrB*, and *fsrC*. By sensing the extracellular accumulation of a peptide lactone encoded at the C terminus of the FsrB protein, the FsrC histidine kinase is activated, subsequently irrigating response regulator FsrA. Inactivation of the *fsr*-controlled gene *gelE* inhibits the biofilm formation [31].

In enterococcal surface proteins (Esps), LPxTG-type surface proteins can covalently immobilize the surface protein to the cell-wall peptidoglycan, involved in cell-to-cell adhesion and biofilm formation [58]. LPxTG-type surface proteins may be partially mediated by the N-terminal moiety of the protein and interacts with additional *E. faecalis*-specific factors to enhance the biofilm formation [58,59]. 

Lipoteichoic acid (LTA) and polysaccharide are also involved in biofilm formation via interactions with environmental molecules. In *E. faecalis*, the *dltABCD* operon is required to obtain D-alanylation of LTA, which is an essential constituent of the Gram-positive bacterial cell wall [60]. The deletion of the *dltA* gene induces the absence of D-alanine in the LTA and a stronger negative net charge on the bacterial cell surface. D-alanine esters as positively charged groups can modulate negatively charged teichoic acids. Subsequently, the biofilm formation was significantly decreased in *E. faecalis* with reduced adherence to epithelial cells, while the susceptibility to cationic antimicrobial peptides was increased [61,62]. 

Enterococcal binding substance (EBS), a kind of cognate receptor for the aggregation substance, such as cell wall LTA, is necessary for mating complexes in biofilm formation [59,63]. Aggregation substances (ASs), such as Asa1, Asp1, and Acs10, promote the conjugation by directing bacterial aggregation, enables cell–cell contact between donor and recipient strains, and increases the *E. faecalis* virulence [64,65]. In a dose-dependent manner, Asc10 with an LTA aggregation domain is located near the N-terminal of *E. Faecalis* LTA [66]. The AS protein is comprised of an N-terminal domain (output signal), a variable region, a central domain (responsible for aggregation), and two Arg-Gly-Asp (RGD) motifs (implicate in binding host cell integrins) [66,67,68]. The expression of the AS protein can be induced by the peptide pheromone, which is secreted from plasmid-free recipient cells, or can be induced by host factors that may act by shifting the effective ratios of endogenous cCF10/iCF10 [65,69,70,71,72,73,74].

The *E. faecalis* WalRK system regulates the various metabolisms including cell wall construction, osmotic protection, biofilm formation, and drug resistance, which are significantly related to bacterial pathogenicity [39]. Furthermore, the drug resistance in *E. faecalis* infection is worrisome, and even some *Enterococcus* spp. are completely resistant to all antimicrobial drug regimens [12,75]. The regulation of *E. faecalis* by the WalRK system includes the physiological metabolisms, biofilm and cell wall formation, drug resistance, and immunity reaction with the host. Considering the WalRK regulations above, it as a novel target can be developed and available for infection disease, especially for the multidrug-resistant vancomycin-resistant Enterococci (VRE). 

Histidine kinase WalK is a transmembrane protein that senses external signals and possesses kinase, phosphotransferase, and phosphatase activities, while the response regulator WalR contains a functional domain (REC) that accepts a phosphate group for autophosphorylation. The kinase ATP-binding domain (HATPase_c) of WalK is stimulated and activated by extracellular signals and then causes phosphorylation at the histidine site of WalK (HisKA domain). The phosphoryl group is first transmitted from the phosphorylated WalK and then to the aspartic acid residue of WalR, leading to conformational change and exposure of the DNA-binding site. Phosphorylated WalR then binds the DNA promoter motif and is involved in transcriptional regulation of bacteria metabolism [48]. In our previous study [76], an antisense *walR* negatively regulates the *walR* expression, leading to the biofilm formation reduction and extracellular polysaccharide synthesis, significantly decreasing the pathogenicity of *E. faecalis*. The roles of WalRK in biofilm formation attracts increasing interest on a potential target of antibacterial and antibiofilm [77]. However, the mechanism of WalRK modulating biofilm formation remains largely unrevealed [78].

### 3.2. Antibiotic Resistance of Enterococcus faecalis

Antibiotic-resistant *E. faecalis* is of concern and contributes to difficult-to-treat nosocomial infections [79]. The main reasons for multidrug-resistant enterococci emergence include intrinsic resistance to antimicrobial agents such as beta lactams and aminoglycosides, and mobile elements acquired resistance against glycopeptides, quinolones, tetracyclines, and streptogramin [80]. The acquired *vanA* and *vanB* genotypes are the most common glycopeptide resistance in *E. faecalis* and are regulated by the VanRS and VanRBSB TCSs [81]. The vancomycin resistance operon modifies the vancomycin-binding target by synthesis of peptidoglycan precursors that terminate in D-lactate or D-serine. Instead of normal D-alanine sidechain terminus, the modified vancomycin resistance operon significantly decreases the affinity vancomycin on *E. faecalis* [82]. With the evolution of resistance, the last-resort antibiotics, such as daptomycin and linezolid, for multidrug-resistant Enterococcal infections are limited [79]. 

*E. faecalis* produces a specific penicillin-binding protein (PBP5) that mediates high-level intrinsic resistance to the cephalosporin class of β-lactam antibiotics. Comenge et al. first described that the regulatory CroRS TCS is essential for PBP5-mediated β-lactam resistance [17]. In further dissection of the CroRS TCS, a noncognate histidine kinase CisS is capable of influencing CroR to mediate resistance to cell wall stresses such as cephalosporins and glycopeptides. The cross-talk of CroRS and CisRS responds to the vancomycin [83]. Vancomycin is the only identified stimuli for CisRS thus far and CisRS can also interact with VanRS for resistance [84]. 

Daptomycin (DAP) is a lipopeptide antibiotic with potent in vitro bactericidal activity against VRE [85]. By binding with calcium, DAP can form a cationic moiety and disrupts membranes to play its potential antimicrobial role [86]. However, reports of DAP-insensitive enterococci strains have become increasingly common [86]. Tran et al. identified the resistance of *Enterococcus faecium* (*E. Faecium*) to daptomycin associated with YycFG system mutation. In addition, this resistance mechanism was also related with the LiaFSR system in *E. faecium* [49]. The LiaFSR three-component system controls the cell envelope stress response of *E. Faecalis* and its mutation results in the resistance of daptomycin [87]. *E. Faecalis* and *E. Faecium* both belong to Enterococcus, and they share a great similarity in gene composition. From this point, it may be inferred that the YycFG system of *E. Faecalis* also plays a role in DAP resistance. 

The two-component systems in bacteria can cross-talk with each other to regulate a variety of cellular processes, including biofilm formation, virulence expression, and drug resistance [26]. For Gram-positive bacteria, the relationship between the WalRK TCS and other TCSs such as VanSB, CovRS, and SaeSR has been explored. In *Streptococcus mutans* (*S. mutans*), VicR and CovR can both regulate a group of genes of extracellular polysaccharides synthesis. However, VicR positively regulates these genes in most cases, while CovR modulates inversely [88]. For *S. aureus*, the VicRK TCS can activate the SaeSR TCS to regulate virulence factors [89]. In *E. faecalis*, VanSB can phosphorylate VicR from *S. pneumoniae* and raise a species cross-talking [6,26]. However, there is rare evidence suggesting interaction between WalRK and other TCSs such as VanSR in *E. faecalis*.

### 3.3. Regulatory Roles of the WalRK TCS in Response to Host Immunity

TCSs in pathogenic bacteria can also adjust responsible immunity activities and infect microenvironments in hosts. For *S. aureus*, WalRK positively regulates virulence genes through a virulence factor regulation system SaeSR TCS, promoting the polymorphonuclear leukocytes (PMNs) lysis and playing an essential role in innate immunity evasion [90]. Additionally, adaptive immunity can enhance the effect of innate immune cells and influence host susceptibility to microorganism invaders [91]. By modulating the virulence expression, the evasion mechanisms from the adaptive immune system are developed [89,92]. In *S. aureus*, a second immunoglobulin binding protein (Sbi) is secreted as a virulence factor, binding with the Fc portion of IgG, and interferes with host adaptive immune responses. In a previous study, WalRK positively regulated biofilms aggregation and virulence genes expression, controlling the progress of the infection condition in the host [93]. However, the specific mechanism of the WalRK TCS on modulating the interaction of the host and *E. faecalis* needs to be further explored.

## 4. Chemical Compounds Act on WalRK to Inhibit the Pathogenicity of Bacteria

Due to the important role of WalRK in Gram-positive bacteria such as *E. faecalis*, *S. aureus*, *S. epidermidis,* and *S. mutans* [94,95,96,97], many attempts were applied on WalRK to inhibit pathogenicity [94].

Clinically, iodoform is widely used as a disinfectant. It has a strong effect on aerobic Gram-positive bacteria and can enhance antibacterial activity against *E. faecalis* when combined with calcium hydroxide [98]. Therefore, it is used in dental treatment, and can be used for the treatment of periapical periodontitis when in combination with calcium hydroxide [99]. However, because of its tendency to cause allergic reactions and cytotoxicity to epithelial cells and macrophages, new drugs need to be developed [100].

### 4.1. Antibiotics-Based Agents on WalRK

Many studies are committed to looking for antibacterial substances from microorganism, such as actinomycin, a good natural antibiotic derived from Actinomycetes.

The autophosphorylation of WalK is an essential step for bacterial pathogenicity. An actinomycin metabolite, waldiomycin, belongs to the family of anguclin antibiotics, which is similar to dioxin in structure, and it can affect the expression of related genes on cell wall metabolism, cell proliferation, and aggregation. Specifically, this inhibitor was found to play a role in inhibiting the function of WalK phosphorylation, attributed to the antibacterial effects mentioned above [94,96]. Subsequently, this waldiomycin has an antibacterial activity against Gram-positive bacteria, including methicillin-resistant S. aureus (MRSA) and *B. subtilis*. Okada et al. pointed out that by binding to the WalK cytoplasmic domain, walkmycin B, which was the main product of *Streptomyces sp.* (MK632-100F11), inhibited WalK phosphorylation [101]. The H-box region is an essential motif of WalK, and Kato et al. found that waldiomycin interfered with various cellular processes of *B. subtilis* by targeting the H-box region of WalK and other HK [102].

**Figure 1:**
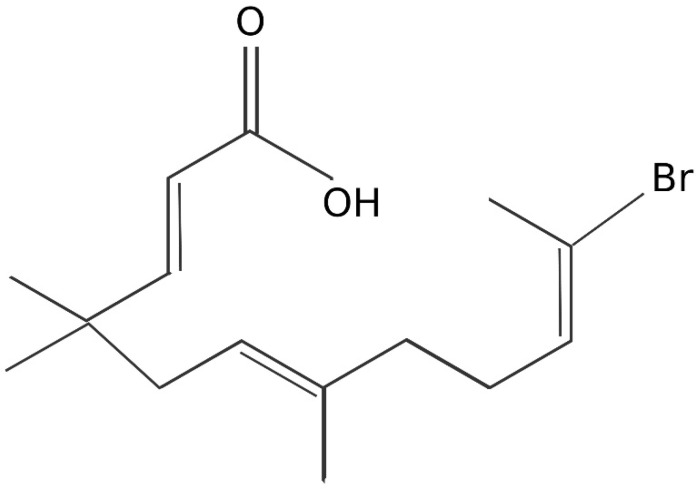
Structure of Zerumbone Ring Opening Derivative 2.

Zerumbone1 is a kind of monocyclic sesquiterpene whose derivatives have high biological activities, including its ring opening derivative 2 (Figure 1). Kitayama et al. pointed out that Zerumbone ring opening derivative 2 can inhibit the autophosphorylation of WalK in *B. subtilis*, while it cannot inhibit drug-resistant bacteria, such as MRSA and VRE [103].

At the same time, signermycin B was found in a temperature-sensitive *B. subtilis* walR mutant (CNM2000) [104]. The signermycin B can inhibit self-phosphorylation by targeting the conserved dimer domain of WalK and inhibit the activity of WalR to hinder the function of the walKR system, achieving an antibacterial effect. 

As the existence of *E. faecalis* biofilm has greatly improved the resistance of *E. faecalis* to existing antibiotic therapy, Lin et al. investigated the effect of derivatives from a kind of probiotic named *B. subtilis* natto on the *E. faecalis* biofilm formation [105]. Natto treatment showed downregulation of the *E. faecalis* walRK system transcription, which resulted in peptidoglycan and glycolipid biosynthesis inhibition and a reduction in extracellular polysaccharide synthesis. These studies showed that *B. subtilis* natto may help to control the biofilm of *E. faecalis*, and then affect the drug resistance of *E. faecalis.*

### 4.2. Novel Screened Antibacterial Substances on WalRK

Recently, many novel antibacterial substances have been developed via high-throughput screening systems with a high efficiency. 

**Figure 2:**
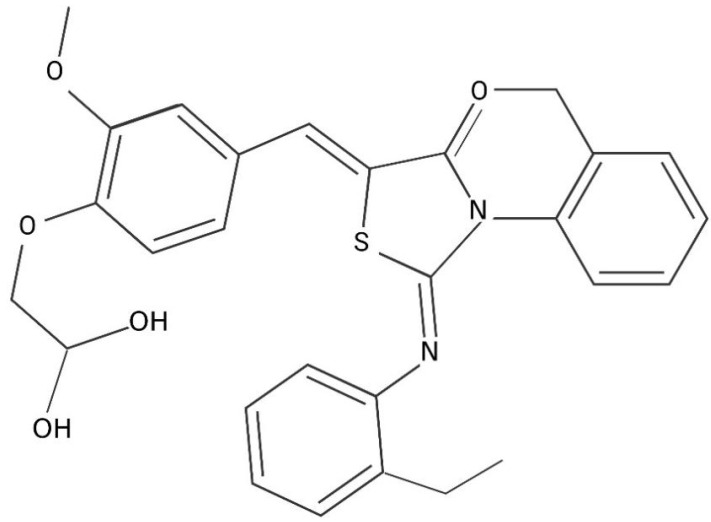
Structure of Thiazolidinone Analogs Compound 2.

Qin et al. verified a series of novel inhibitors on the *S. epidermidis* WalK protein by using structure-based virtual screening (SBVs) in a small-molecule lead compound library [106]. There, seven inhibitors belonging to four different chemical structures were identified: three thiazolidinone analogs (compounds 2, 5, and 7), two benzamide analogs (compounds 1 and 3), a furan derivative (compound 4), and a pyrimidine ketone derivative (compound 6). By replacing different functional groups in Compound 2 (C27H28O5N2S) (Figure 2), a series of derivatives were designed, such as H2-38, H2-39, H2-74, and H2-81 (Table 1) [107]. Those derivatives as new antibacterial agents targeting WalK can effectively eradicate against clinical Staphylococcus isolates such as multidrug-resistant *Staphylococcus* spp. and *S. aureus.* H2-60 (C27H16F2O2N3S2) and H2-81(C27H17FO2N3S2) are two thiazolidinone derivatives. Both derivatives are proved to have an effect on WalK. Biofilm has greatly improved the resistance of *E. faecalis* to existing antibiotic therapy. In *E. faecalis*, Chen et al. identified two derivatives that play a role against biofilm production by repression of WalK kinase phosphorylation activity [108]. When combined with traditional antibiotics such as daptomycin, H2-60 can even synergistically enhance the sensitivity of *E. faecalis* than single application.

In other species, such as *Streptococcus agalactiae*, the verified H2-60 and H2-81 can significantly inhibit biofilm formation and bacterial growth [109]. Furuta et al. screened and identified a compound I-8-15 (1-dodecyl-2-isopropyl imidazole), which can specifically inhibit the WalK of *S. aureus*, resulting in conformational changes and inhibiting dimerization of WalK [110]. By this mechanism, I-8-15 showed a significant antibacterial activity against MRSA and VRE. 

In addition, Gotoh et al. also identified wallycin A (4-methoxy-1-naphthol) and wallycin B (1,6-dimethyl-3-[4-(trifluoromethyl) phenyl] pyrimido [5,4-e][1,2,4]triazine-5, 7-dione) compounds through the high-throughput screening system [95]. IclR is a regulatory protein containing the N-terminal DNA binding domain of the wing helix trans helix motif and the C-terminal regulatory domain of the protein, and the binding of signal molecules to the C-terminal domain of IclR is considered to inhibit the transcription of target genes by regulating DNA binding activity or the polymerization of IclR. By analysis, it was proved that these two compounds can inhibit the phosphate transfer from P-WalKtru to WalR. As a result, they can inactivate the IclR-WalR chimeric repressor, as walR inhibitors.

### 4.3. Nucleic-Acid-Based Interfering walRK for Treatment

Antisense RNA (asRNA) is a single-stranded RNA reverse complement to the targeted mRNA. It can inhibit the translation or transcription of target mRNA by recruiting the RNase to degrade RNA duplex structures. Using this property, RNA interference technology is developed to inhibit the transcription of target genes to affect physiological functions [111].

*E. faecalis* is the main pathogen of persistent periapical periodontitis. The *E. faecalis* biofilm formation is related to the WalRK modulation. How to inhibit WalRK expression and reduce biofilm formation attracts a prospective research topic. Our previous study identified an endogenous antisense *walR* (ASwalR) inhibiting *walR* expression [112]. With ASwalR interference, the biofilm formation of *E. faecalis* is significantly reduced and sensitivity to traditional chlorhexidine is increased. To further improve the efficiency of ASwalR, a graphene-oxide (GO)–polyethyleneimine (PEI)-based transformation strategy is applied, indicating that it can be used as a potential supplementary therapy for infection treatment [113].

### 4.4. Bacteriophage Therapy

Phage is the general name of viruses that infect microorganisms such as bacteria, fungi, algae, actinomycetes, and spirochetes. The size of phages is generally small, with an incomplete cell structure, and only contains a single nucleic acid. In addition, some of the phages can cause the lysis of host bacteria. By nucleic acid invasion and replication, the phages can kill bacteria as a novel treatment mode. Unlike traditional antibiotics, phages replicate themselves in the bacteria, and this repeating process effectively amplifies its antibacterial efficiencies. Because of antibiotic resistance and the fact that antibiotics may destroy the microbial balance in the body, phage therapy is less likely to develop resistance and is also highly microbial-specific [114].

However, phage therapy has some corresponding disadvantages including the specificity to target bacteria, easily producing corresponding tolerance under selection, being contaminated by bacteria used for culture, and rapidly being removed from the body and other unclear efficacies [115]. Therefore, many studies have been performed to improve the above shortcomings.

To overcome short duration, Merril et al. screened a mutation of the main phage head protein E in capsid proteins of phage K and phage P22, in which a long-half-life phage was acquired [116]. Meanwhile, chemical modification with polyethylene glycol (PEG) can significantly improve the cycle half-life of phages by reducing the release level of helper-T-cell-type-Ⅰ-related cytokines (IFN-γ and IL-6) [117].

The biofilm formation significantly decreases the sensitivity of bacteria on phages. To strengthen the effect of phages, Lu modified a phage to encode enzymes [118]. Enzymes can hydrolyze extracellular polymeric substances and help the phage to bind to the receptor. Antibiotic efflux pumps are the essential resistance mechanisms. The combination of antibiotic treatment and bacteriophages acting on the expression of antibiotic efflux pumps can make bacteria more sensitive to antibiotics [119,120].

In *E. faecalis*, phages can potentially be a genetic scheme for treatment. Duerkop et al. found sewage phages in urban sewage [121]. Through phage infection proteins for Enterococcus faecalis (PIPEF), sewage phages inhibit the colonization of *E. faecalis* in the intestine, playing a therapeutic role.

Therefore, the phage therapy is also expected as a good scheme for the infection treatment. We proposed a hypothetical treatment, which integrates the antisense fragment of the *walRK* gene or inhibitor encoding the WalRK protein on the phage genome specific to *E. faecalis* and modified by PEG, and then injects it into the human body to treat *E. faecalis* infection to inhibit the biofilm formation, cooperating with corresponding antibiotic treatment.

## 5. Conclusions

Biofilm formation in *E. faecalis* induces a high adaption to the environment and causes severe infections. Many factors including polysaccharides as well as autolysis, proteases, and eDNA regulate biofilm formation. These factors are regulated by several important regulatory systems involving the TCSs. The highly conserved WalRK, one of 17 TCSs, is the only essential TCS in *E. faecalis*. In addition, various metabolisms, including cell wall construction, drug resistance, and interactions between regulatory systems and resistance to the host immune system, can be regulated by the WalRK system. Therefore, WalRK has been identified as a key target for the infection control. At present, novel antibiotics, screened antibacterial substances, and nucleic-acid-based agents interfering on WalRK phosphorylation or expression have been investigated for infection treatment. Meanwhile, promising phage therapy, a potentially genetic scheme, shows a good inhibition on bacterial growth. Therefore, suppressing the action of WalRK on bacterial infection with programed phages such as antisense fragment integration will be a topic of great interest.

## Figures and Tables

**Table 1:** Chemical composition of Thiazolidinone Analogs Compound 2 Derivatives.

Thiazolidinone Analogues Compound 2 Derivatives	
Name	Chemical Formula
H2-38	3-{5-{{3-(4-chlorophenyl)-2-[(4-chlorophenyl)imino]-4-oxothiazolidin-5-ylidene}methyl}furan-2-yl}benzoic acid
H2-39	4-{5-{{3-(4-chlorophenyl)-2-[(4-chlorophenyl)imino]-4-oxothiazolidin-5-ylidene}methyl}furan-2-yl}benzoic acid
H2-74	2-{4-{{3-(4-chlorophenyl)-2-[(4-chlorophenyl)imino]-4-oxothiazolidin-5-ylidene}methyl}phenoxy}acetic acid
H2-81	4-{5-{{3-(4-fluorophenyl)-2-[(4-phenyl)imino]-4-oxothiazolidin-5-ylidene}methyl}thiophene-2-yl} benzoic acid

## Data Availability

Not applicable.
